# Towards responsible research assessment: How to reward research quality

**DOI:** 10.1371/journal.pbio.3002553

**Published:** 2024-02-26

**Authors:** Anne Gärtner, Daniel Leising, Felix D. Schönbrodt

**Affiliations:** 1 Faculty of Psychology, Technische Universität Dresden, Dresden, Germany; 2 Department of Psychology, Ludwig-Maximilians-Universität München, München, Germany

## Abstract

Researchers would be more willing to prioritize research quality over quantity if the incentive structure of the academic system aligned with this goal. The winner of a 2023 Einstein Foundation Award for Promoting Quality in Research explains how they rose to this challenge.

There seems to be a growing consensus in academia that an individual’s scientific achievements should no longer be evaluated with mainly quantitative indicators (such as the number of publications, the journal impact factor, or *h*-index), but that greater weight should be given to the quality, transparency, reproducibility, and innovative strength of their scientific work. This shift comes in response to the realization that the current system of research assessment, while efficient in some respects, may inadvertently encourage behaviors that hinder the advancement of knowledge. Several initiatives already exist that aim to address this challenge. For example, back as in 2012, the San Francisco Declaration on Research Assessment (DORA) called for an end to the use of invalid quantitative indicators (e.g., the journal impact factor) and was signed not only by important third-party funding bodies but also by scientific associations. Recently, DORA launched Reformscape, an online tool to explore examples of how to implement responsible research assessment for hiring, promotion, and tenure in institutions and to share approaches from different fields and institutions. Within Europe, some of the institutions that signed DORA also joined the Coalition for Advancing Research Assessment (CoARA), which aims to fundamentally reform the ways in which research performance is evaluated.

At the same time, the practice of university appointment procedures shows that easily measurable quantitative indicators continue to be prioritized, while the assessment of the quality, rigor, reliability, robustness, transparency, and innovativeness of candidates’ scientific work seem to be less important. Criteria catalogues in appointment procedures represent implicit incentive systems that reward certain activities, such as publishing many articles, more than, for example, research transparency, reproducibility, or further training in leadership and management skills. These incentive systems can therefore have undesirable effects on the entire science system [1,2], especially if mechanisms of quality control (e.g., the peer review system) and self-correction of the system are not sufficiently effective [3].

The strong weighting of indicators of pure quantity of research output is problematic in the assessment and prediction of excellent scientific performance due to their questionable validity [4–7]. Moreover, the correlation between journal rank (as measured by journal impact factor) and the methodological quality of papers published in a journal is low or even negative [8]. Academia is a highly competitive work environment, and incentives in hiring and promotion processes thus have a direct impact on the behavior of individuals, including the willingness to engage in questionable research practices, which may, in turn, jeopardize the robustness of published findings [9].

Therefore, our initiative for “Responsible Research Assessment” seeks to address this problem by proposing a more holistic, nuanced, and actionable evaluation framework [1,2,10] called RESQUE (Research Quality Evaluation). One of us (Anne Gärtner) was awarded the 2023 Early Career Award for Promoting Quality in Research from the Einstein Foundation Berlin for work on a project as part of this initiative. In line with the “Recognition and Rewards” program by Dutch research organizations and a position paper by the League of European Research Universities, we previously proposed 4 guiding principles: that diverse academic contributions (beyond journal articles) should be valued, including data set publications and research software development; quantitative indicators must be valid and used responsibly; methodological rigor, impact, and quantity should be considered independently in evaluating research; and quality should be valued over impact and quantity. While these fundamental principles overlap considerably with those of other initiatives (such as DORA and CoARA), the RESQUE system is (among) the first to actually present a set of specific evaluation criteria for research output. A free online assessment tool is currently under development.

A working group appointed by the German Psychological Society published a concrete proposal in a series of articles [1,2,10] and received more than 40 commentaries from the academic community in response. These now inform the revision of the proposal, which is currently underway. In the meantime, the broader project has turned into a community-driven effort. Recommendations are being revised and extended in multiple bottom-up working groups, and disciplinary sections of the academic society started to discuss and to work on field-specific expansion packs. The results will also be fed into the broader CoARA process.

The first concrete proposal (published as a preprint in 2022) includes a 2-stage evaluation process that combines the objectivity and efficiency of using metric indicators (Stage 1) with an in-depth, discursive evaluation of actual research content (Stage 2) [10]. Arguing in favor of broadening the range of relevant research contributions, our proposal introduces quality criteria for research articles, datasets, and research software. These criteria emphasize the methodological rigor of such contributions (given that methodology defines scientific rigor). Some of the relevant questions are: was the research preregistered; are data and code provided in an openly accessible, comprehensive, and reusable way (e.g., FAIR format); can the research be replicated and computational results be independently reproduced; and do theoretical formulations adhere to the principles of formal logic?

These criteria and the resulting multidimensional research profile (for an example, see Fig 1) are to be used in Phase 1 of the evaluation process to filter out applicants with insufficient methodological rigor, too low productivity, and which lack the necessary criteria from other types of academic contributions such as teaching (e.g., by establishing minimum thresholds). Candidates that pass this negative selection are to be considered for the short list.

**Fig 1:**
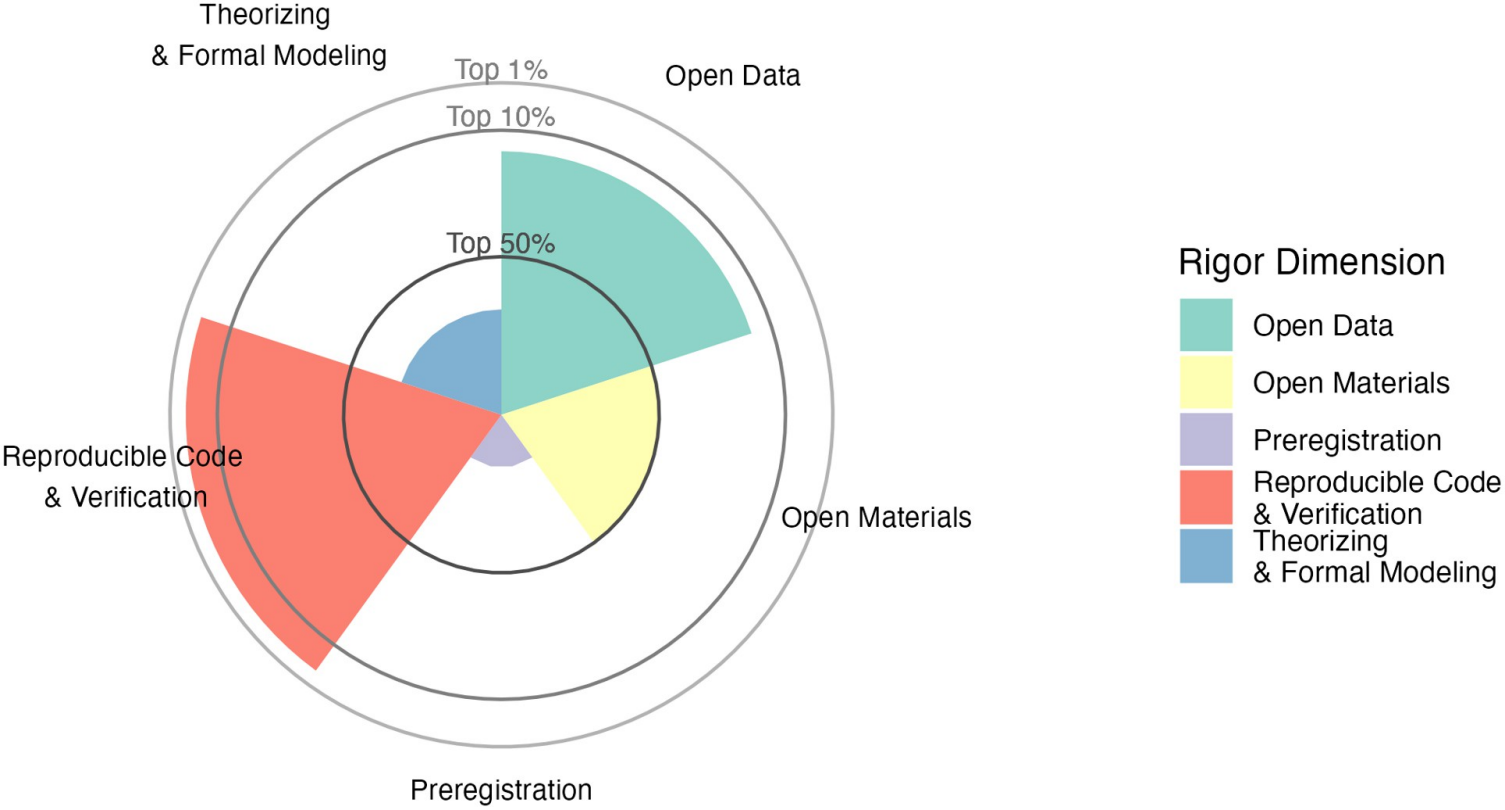
Methodological rigor profile. Exemplary output from the RESQUE tool, showing a summary of the methodological quality of an applicant’s work.

Phase 2 of the evaluation process focuses more on the actual content and merits of a candidate’s research, no longer making much use of metrics. Here, other criteria such as ambition, relevance, innovation, and creativity are to be evaluated (positive selection). This requires in-depth discussions of an applicant’s work with them, among committee members, and with external reviewers.

A free online tool that supports Phase 1 assessments of the methodological rigor of applicants’ research outputs is now available. Quality criteria for other types of academic contributions (e.g., teaching, leadership, governance, societal impact) have yet to be developed and tested. The RESQUE system was initially developed within and for psychology, but we are confident that it may be applicable in other fields (such as biology) as well. This is because many of the practices that may be used to help promote quality (such as open data or preregistration) are equally relevant to most branches of empirical science. However, some field-specific adaptations may still be necessary. We would like to expressly encourage our colleagues from other fields to get in touch with us and begin working together to enable this transfer.

The shift away from metrics of publication quantity in hiring and promotion procedures could ultimately become a blueprint for the entire academic system and help inform the distribution of research funding, scholarships, and awards. By shifting the focus towards quality, we can build a research culture that not only rewards genuine contributions but also paves the way for a more robust and impactful scientific future.

## Abbreviations

CoARA: Coalition for Advancing Research Assessment
DORA: Declaration on Research Assessment
RESQUE: Research Quality Evaluation
